# Are Faster Participants Always Faster? Assessing Reliability of Participants’ Mean Response Speed in Picture Naming

**DOI:** 10.5334/joc.337

**Published:** 2024-01-11

**Authors:** Pamela Fuhrmeister, Shereen Elbuy, Audrey Bürki

**Affiliations:** 1 Universität Potsdam, Germany

**Keywords:** language production, picture naming, reliability, individual differences

## Abstract

Studies of language production often make use of picture naming tasks to investigate the cognitive processes involved in speaking, and many of these studies report a wide range of individual variability in how long speakers need to prepare the name of a picture. It has been assumed that this variability can be linked to inter-individual differences in cognitive skills or abilities (e.g., attention or working memory); therefore, several studies have tried to explain variability in language production tasks by correlating production measures with scores on cognitive tests. This approach, however, relies on the assumption that participants are reliable over time in their picture naming speed (i.e., that faster speakers are consistently fast). The current study explicitly tested this assumption by asking participants to complete a simple picture naming task twice with one to two weeks in between sessions. In one experiment, we show that picture naming speed has excellent within-task reliability and good test-retest reliability, at least when participants perform the same task in both sessions. In a second experiment with slight task variations across sessions (a speeded and non-speeded picture naming task), we replicated the high split-half reliability and found moderate consistency over tasks. These findings are as predicted under the assumption that the speed of initiating responses for speech production is an intrinsic property or capacity of an individual. We additionally discuss the consequences of these results for the statistical power of correlational designs.

In psycholinguistic research on language production processes, studies tend to examine behavior at the group level. In the present study, we focus on word production. A measure of choice in this field is the production latency, or time required to prepare a word for production following the presentation of a stimulus, often a picture (e.g., Alario et al., 2004; Costa, Strijkers, Martin, & Thierry, 2009; Fargier & Laganaro, 2019; Fieder, Wartenburger, & Rahman, 2019; Laganaro, Valente, & Perret, 2012; Meyer, 1996; Pinet, Ziegler, & Alario, 2016; Rabovsky, Schad, & Rahman, 2016; Roelofs, 2004). In many of these studies, production latencies from different experimental conditions are compared (e.g., with and without priming, high and low frequency), but variability in average speed across participants is of little interest: Participants are treated as a random factor in the analysis to ensure that experimental effects generalize to all participants, irrespective of their speed. Several effects found in language production studies (e.g., word frequency, age of acquisition, priming effects) have been reported and have been shown to be replicable across groups and studies, suggesting that naming latencies are a good index of language production processes. Yet, individual speakers show a wide range of variability in the time needed to prepare spoken words.

The extent of inter-individual variability in language production has for instance been reported for picture naming tasks (e.g., Bürki, 2017; Laganaro et al., 2012; Valente, Bürki, & Laganaro, 2014). In contrast to the dominant approach, several recent studies have focused on this variability with the idea that inter-individual differences in picture naming speed can inform our understanding of the architecture of the language production system and how it relates to non-linguistic abilities. Some of these studies have for instance taken a correlational approach to investigate relationships between participants’ picture naming speed and performance on cognitive tasks. For example, correlations between measures of sustained attention and picture naming speed have been reported (Jongman, 2017; Jongman, Meyer, & Roelofs, 2015; Jongman, Roelofs, & Meyer, 2015). Other studies have observed relationships between working memory measures and picture naming latencies (Piai & Roelofs, 2013; Shao, Roelofs, & Meyer, 2012; but see Klaus & Schriefers, 2018), and a few studies have found that inhibition skills are related to picture naming speed (Lorenz, Zwitserlood, Regel, & Abdel Rahman, 2019; Shao, Meyer, & Roelofs, 2013; Sikora, Roelofs, Hermans, & Knoors, 2016).

The hypothesis that (picture) naming speed relates to participants’ cognitive abilities relies on the assumption that relatively faster speakers are relatively faster each time they are tested. It further implies that differences in naming speed are not specific to the details of the task at hand (e.g., timing of individual trials). The aim of the present study is precisely to assess this reliability for picture naming latencies. We first assess the reliability of individual differences in picture naming speed within the experimental session and over time. We then test whether participants are still ranked by mean naming speed in a similar way across different manipulations of the same task.

In the remainder of this introduction, we first briefly discuss the concept of reliability in the context of inter-individual differences and how it can be assessed. We then discuss the importance of establishing the reliability (or lack thereof) of participants production speed for language production research. Finally, we introduce the current study in some detail.

## Reliability and inter-individual differences

Broadly, the term reliability refers to the consistency or trustworthiness of a measure (Urbina, 2014), although the term has slightly different meanings in different contexts. For example, in studies comparing performance on a task between groups or experimental conditions, the term reliability is sometimes used to describe an experimental effect that is replicable across different samples of participants (Hedge, Powell, & Sumner, 2018). In studies focusing on inter-individual differences, scores on a test or task are said to be reliable if a participant’s performance, measured on different occasions, is highly consistent. In these studies, it is important to use measures with low measurement error, or high reliability (Parsons, Kruijt, & Fox, 2019) because such measures will produce consistent inter-individual differences. That is, if the performance of participant A is better than that of participant B on a given day or for a subset of trials, participant A will be better than participant B on a different set of trials or when tested on a different day. In certain subfields of psychology that have traditionally focused on individual differences (e.g., differential psychology), reporting reliability is standard, for example in personality research (Viswesvaran & Ones, 2000) or intelligence testing (Donders, 1997). However, this is not often the case in cognitive psychology (Parsons et al., 2019).

Reliability of participant scores can be measured in different ways. A common way of assessing reliability within an experimental session is to correlate two halves of the data (e.g., even vs. odd trials, first half vs. second half), which is referred to as split-half reliability (Parsons et al., 2019; Urbina, 2014). The term test-retest reliability is often used to refer to reliability over time (e.g., at different sessions, Urbina, 2014). To estimate test-retest reliability of a given measure, the same participants are tested two (or more) times and the correlation between their performance at different time points is computed (Urbina, 2014). If inter-individual differences are reliable, correlations between participants’ performance across trials or sessions should be high. To reiterate, saying that a measure is reliable amounts to saying that inter-individual differences are themselves reliable.

An important question in any study of reliability is what evidence is needed to determine whether performance on a task is reliable. For instance, a correlation of .2 could be statistically significant, but a weak correlation that is statistically significant may not be very meaningful when considering the issue of reliability. If a Pearson correlation of .2 was obtained when assessing test-retest reliability for a measure, it means that 96% of the variance remains unexplained. A correlation of .9 would be much more convincing because only 9% of the variance is left unexplained. Though there is not one standard cutoff for an effect size that is considered to be indicative of good reliability, most authors suggest that a measure is reliable enough if the correlation is at least 0.7 or 0.8 (Jhangiani, Chiang, & Price, 2015); however, the standard might be higher for clinical situations (Hedge et al., 2018; Nunnally, 1994; Parsons et al., 2019).

## Reliability of participants’ speed in picture naming

Despite the recent interest in individual differences in word production research, we still lack studies testing the reliability of word production measures. In the present study, we focus specifically on reliability of picture naming measures. The available evidence suggests that picture naming speed is relatively reliable *within an experimental session*. For example, Shao et al. (2013) reported high split-half reliability (correlation of even and odd trials) for mean picture naming speed (*r* = .91) in a picture naming task. In addition, we find a high correlation between even and odd trials in our own picture naming data, *ρ* = 0.97 (95% credible interval [0.93, 1.00], reanalysis of Fuhrmeister, Madec, Lorenz, Elbuy, & Bürki, 2022). Shao et al. (2012) had participants name pictures of objects and actions, and they found a fairly high correlation between participants› mean naming latencies of objects and actions (*r* = .74) and replicated this high correlation (*r* = .86) in a later paper (Shao, Roelofs, Acheson, & Meyer, 2014). This suggests participants are also relatively consistent in their speed of naming two different kinds of stimuli.

Importantly, finding a high correlation *within* an experiment is necessary but not sufficient to conclude that picture naming tasks generate reliable differences across participants. Naming latencies for the different trials of an experiment could be correlated because participants set a goal that is specific to the experimental setting, or feel more or less motivated or alert on a given day due to temporary factors, such as sleep quality and duration the night before, time of day that the experiment took place, or temporary emotional state. Notably, if the consistency of participants’ naming times within an experimental session is due to one of the aforementioned factors, correlations may be expected between naming times and performance on non-linguistic tasks when the participants are tested on the language production and non-linguistic tasks on the same day, within the same session. As a result, these correlations may not reflect individual differences in cognitive skills. If a participant’s speed can partly be explained by their cognitive abilities, we expect naming latencies to also be consistent over time, i.e, when participants are tested on different days.

We further expect that differences across participants will persist when the experimental setting is modified such that it prompts differences in response speed (e.g., shorter inter-stimulus intervals, instructions to respond within a given time window). In picture naming experiments, it is possible that differences across participants arise because they have room to set different goals for the task. If they have ample time, they can select their own pace. For instance, some participants could decide to respond as quickly as possible, while others might prioritize accuracy over speed, or simply select a comfortable response speed given the allotted time. The hypothesis that differences in naming latencies reflect differences in cognitive abilities implicitly assumes that there is limited room for “strategies” or “decisions.”

We mentioned above that several of the studies that examined differences in naming speed across participants used a correlational approach, i.e., correlated naming latencies with performance on a cognitive task. In this context, the reliability of participant naming latencies has crucial methodological implications, in that reliability is directly related to statistical power. When assessing the correlation between two measures, e.g., performance on picture naming and working memory tasks, the reliability of the individual measures constrains the magnitude of the correlation that can be found *between* these measures (Parsons et al., 2019). The number of participants required to detect a correlation between two measures increases when the reliability of these measures decreases (e.g., Hedge et al., 2018, Table 5). Thus, reliability estimates of measures of interest can be used in power calculations to determine the sample size needed to detect effects of a certain magnitude (Parsons et al., 2019). For example, assuming a true correlation of 0.3 between two measures, 133 participants would be necessary to reach the standard significance threshold when the two tasks have a reliability of 0.8; 239 when the reliability is 0.6. Pilot data from our lab shows a correlation of .28 between a measure of attention and naming latencies. Assuming that this number reflects the true effect size, the number of participants required to detect the correlation given a reliability of 0.8 for each task would be 153. If this estimation is correct, sample sizes such as the one we used (n = 45) are likely to be insufficient to detect such correlations. The reliability of some of the measures of cognitive skills that have been used in correlational studies to explain individual differences in picture naming has been tested independently (see e.g., Borgmann, Risko, Stolz, & Besner, 2007 for reliability of the Simon effect; Congdon et al., 2012 for an example involving the stop-signal reaction time task; and see Conway et al., 2005; Klein & Fiss, 1999; Waters & Caplan, 2003 for working memory measures) or reported in the paper along with correlations with picture naming measures (Jongman, Roelofs, et al., 2015). If the reliability of one or more measures entered into a correlation is low, power to detect these correlations suffers, and the probability of Type II errors increases. Precise estimates of reliability of both measures entered into a correlation are therefore necessary to make sure that the required sample size is tested. Hedge et al. (2018) even describe reliability of a measure as “a prerequisite to effective correlational research.”

## Current study

The current study consists of two experiments. Each experiment tests a group of participants at two different sessions that occur between 7 and 14 days apart. Experiment 1 tests split-half reliability and test-retest reliability of simple picture naming (i.e., naming of bare nouns). The same task was used for both sessions. The implementation of the task, including timing, mimics that of a standard picture naming task. Participants were presented with a picture and had 3000 ms to provide their response.

Evidence of reliability within and between sessions would be in line with the assumption that cognitive abilities of an individual impact picture naming speed. However, if picture naming speed is not reliable over time, this would suggest that inter-individual differences in naming speed do not index general differences in cognitive abilities. This would not mean that previous studies that have found correlations between cognitive skills and picture naming speed are not informative; it may simply limit the extent to which we can generalize these findings. For instance, these correlations might not necessarily indicate that individuals who have better attention or inhibition skills are faster speakers; rather, they may mean that the amount of attention or inhibition applied within a specific task is more relevant for picture naming speed than attention or inhibition abilities measured from an independent task.

In Experiment 2, we test the correlation between participants’ naming speed on a picture naming task, in which we manipulate the conditions under which participants name the pictures. One condition is a simple picture naming task like in Experiment 1, and the other is a speeded naming task, in which participants have a limited amount of time to name the picture (i.e., a response deadline). Previous studies have consistently shown that picture or word naming speed is faster with a response deadline, at least at the group level (Damian & Dumay, 2007; Kello, Plaut, & MacWhinney, 2000). With this manipulation, we can test whether participants are ranked similarly in speed with or without a response deadline. Relatedly, we can examine whether participants may be engaging in a speed-accuracy trade-off strategy (Heitz, 2014).

In word production studies using a response deadline, the evidence of a speed-accuracy trade-off is mixed. For example, Kello et al. (2000) found similar error rates on speeded and non-speeded versions of the Stroop task with naming responses. Starreveld and La Heij (1999) and Damian & Dumay (2007, in one out of three experiments) found a speed-accuracy tradeoff in picture naming experiments, but in both these experiments, participants were specifically instructed to make errors to prioritize speed. Moreover, in these experiments, the speed-accuracy trade-off was examined at the group level. To our knowledge, no studies have looked at individual differences in speed-accuracy trade-offs in picture naming tasks to determine whether individual participants are engaging in strategies to choose their picture naming speed. If they do, then the observed inter-individual differences in mean naming speed could in part be due to participant-specific decisions rather than individual differences in cognitive abilities. If participants are engaging in such strategies or picking a specific tempo for the task, we expect that inter-individual differences will be less reliable when measured between picture naming tasks that vary in timing.

## Experiment 1

Experiment 1 tested within- and between-session (i.e., split-half and test-retest) reliability of participants’ picture naming speed over two sessions (7–14 days apart) using a simple picture naming task.

### Methods

#### Participants

Participants were recruited through the online platform Prolific (www.prolific.co), and the study was advertised to native speakers of British English with no history of reading or language disorders. We recruited participants until we had usable data from 50 participants (78 total) because we could reasonably pre-process that amount of data (per experiment) with our current lab resources. Participants were excluded if they did not complete both sessions (*n* = 20), the data were not recorded due to technical errors (*n* = 7), or the recording quality was so low that we were unable to detect the onset of the vocal responses (*n* = 1). We additionally planned to exclude participants if there was an obvious indication they did not follow instructions, for example, if we heard from the recording that they were listening to music, talking to other people, or eating during the experiment. Fairs and Strijkers (2021) did a recent picture naming study online and found that some participants kept the experiment running in order to get paid for it but did not actually do it. In order to eliminate participants who did not perform the experiment in good faith, we required participants to reach at least 60% accuracy in naming the pictures to be included in the analyses. Those who did not reach this threshold in the first session were not allowed to participate in the second session. This was not necessary in the first experiment because all participants who were not excluded for reasons listed above achieved at least 60% accuracy. Participants were excluded prior to any data analysis, and all excluded participants were replaced so that the final sample size was 50. Participants gave informed consent prior to the experiment and were paid €11 per hour. This study was approved by the ethics board of the University of Potsdam.

#### Stimuli

We selected 310 pictures from the Multipic database (Duñabeitia et al., 2018) with the highest name agreement ratings that also had corresponding data for frequency and age of acquisition available in relevant databases. The Multipic database provides freely available, colored drawings of 750 words with norms in several languages (Duñabeitia et al., 2018). It has been used in many picture naming experiments (e.g., Bartolozzi, Jongman, & Meyer, 2021; Borragan, Martin, De Bruin, & Duñabeitia, 2018; Gauvin, Jonen, Choi, McMahon, & Zubicaray, 2018; Zu Wolfsthurn, Robles, & Schiller, 2021), including a recent experiment run online (Fairs & Strijkers, 2021). In cases of duplicate target words for different pictures, one of the pictures was removed and replaced with another. Information on lexical frequency was obtained from the SUBTLEX-UK database (Van Heuven, Mandera, Keuleers, & Brysbaert, 2014), and age of acquisition data was obtained from Kuperman, Stadthagen-Gonzalez, and Brysbaert (2012). The H-index was provided by the database as a measure of name agreement. The H-index takes into consideration how many different names are supplied for the picture as well as the frequency that alternative names are given; a lower H-index indicates higher name agreement. Pictures included in the study had a maximum H-index of 0.52, and at least 88.9% of people gave the modal name when the pictures were normed (Duñabeitia et al., 2018).

We created two different lists from the 310 pictures (155 items each) such that they would be balanced on word frequency, name agreement, and age of acquisition. We chose to equate lists on these three variables because they have been found to be some of the most robust predictors of naming latencies across several studies conducted in several languages (Alario et al., 2004; Cuetos, Ellis, & Alvarez, 1999; Ellis & Morrison, 1998; Snodgrass & Yuditsky, 1996).^1^ Lists can be found in Appendix A. The procedure for creating the balanced lists was as follows: We first obtained values of name agreement, frequency, and age of acquisition for all 310 pictures and z-scored these values. These values formed a feature vector for each stimulus item. We then calculated the cosine similarity of the feature vectors for each unique pair of stimuli and sampled one million random sets of 155 pairings of stimulus items and calculated the mean cosine similarity of all the pairings in each set. We chose the set of 155 pairings with the highest mean cosine similarity for the two lists, as these were the most similar in terms of lexical frequency, name agreement, and age of acquisition (cosine similarity = .24). Descriptive statistics on these measurements from each list can be found in Table 1. Reproducible code for list creation can be found at the OSF repository for this project: https://osf.io/jqmtv/. Participants saw one of the two lists of pictures at each session (5 pictures per list for training items; 150 pictures per list for test items), and the order of list presentation was counterbalanced (i.e., half of the participants saw List 1 for Session 1 and half saw List 1 for Session 2). All participants saw the same 5 pictures in each list for training.

**Table 1:** Mean and standard deviation (SD) of frequency (Zipf scale), age of acquisition (AoA) ratings, and name agreement (H-index) for the words/pictures in each list.


LIST	FREQUENCY MEAN	FREQUENCY SD	AoA MEAN	AoA SD	H-INDEX MEAN	H-INDEX SD

1	4.17	0.48	5.39	1.43	0.13	0.15

2	4.28	0.64	5.48	1.69	0.13	0.17


#### Procedure

**Figure 1:**
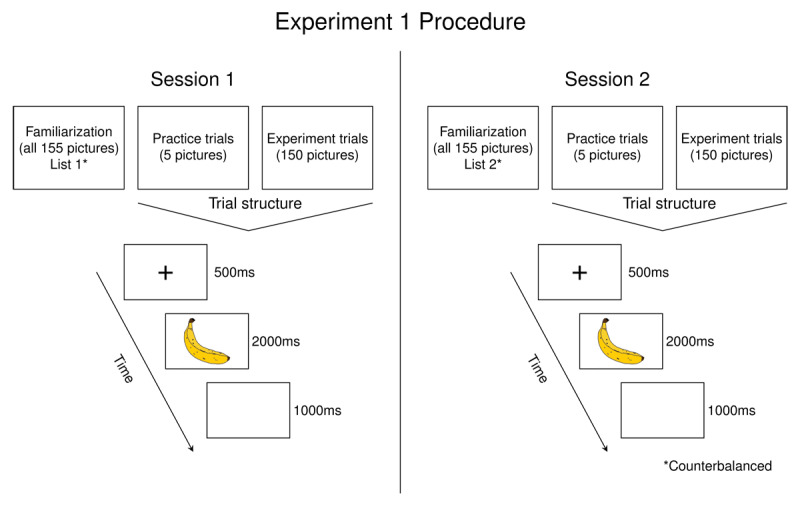
Illustration of procedure for Experiment 1. Participants completed the depicted experiment at two different sessions, each with a different stimulus list. The order of the stimulus list presentation was counterbalanced.

The experiment consisted of two sessions (see Figure 1). The second session took place between 7 and 14 days after the first session to assess reliability of picture naming speed over time. All stimuli were presented online using the experiment presentation software PCIbex (Zehr & Schwarz, 2018).

Participants named each picture in a list (5 practice trials, 150 experimental trials) in a simple picture naming task in each session. At the beginning of each session, participants were familiarized with all of the stimuli by seeing each picture with the printed target word below it on the screen. Participants were asked to study the pictures and were told they will need to recall the name of the pictures for the next part of the experiment.

The picture naming task began with a brief practice phase of five trials, followed by the main part of the task with 150 trials. Each trial began with a fixation cross that appeared in the center of the screen for 500 ms, followed by a picture which appeared for 2000 ms. Then the picture disappeared and participants saw a blank screen for 1000 ms. Vocal responses were recorded from the onset of the picture until the end of the trial. Participants were instructed to name the picture aloud as fast and accurately as they could. All 150 pictures were presented in random order.

#### Planned analyses

##### Data preprocessing

Only trials with correct responses were included in the analyses. Incorrect responses included trials for which participants produced the wrong word (Session 1: *n* = 232, 3.09% of all trials; Session 2: *n* = 215, 2.87% of all trials), exhibited disfluencies (e.g., false starts, Session 1: *n* = 41, 0.55% of all trials; Session 2: *n* = 48, 0.64% of all trials), and trials on which no response was given within 3000 ms (the length of the trial, Session 1: *n* = 93, 1.24% of all trials; Session 2: 50, 0.67% of all trials). We did not filter the data for outliers.^2^ Picture naming latencies were calculated for each trial as the time between the picture onset and the onset of the vocal response; the latter was set manually in Praat (Boersma & Weenink, 2021). The preprocessed data set^3^ and analysis code for all experiments in the study is publicly available at https://osf.io/jqmtv/.

##### Split-half (within-session) and test-retest (between-session) reliability

All analyses were done in R (R Core Team, 2021). To estimate split-half and test-retest reliability, we computed the correlation between response times in each half of the data in each session separately (split-half reliability) or between each session (test-retest reliability). Correlations were computed in Bayesian hierarchical models (the correlation of the random effects) using the package brms (Bürkner, 2017). Correlations of random effects estimated from a hierarchical model more accurately reflect participants’ “true” effects due to shrinkage from the model and because hierarchical models take trial noise and item variability into account (Chen et al., 2021; Haines et al., 2020; Rouder & Haaf, 2019). We chose this approach as opposed to an intraclass or Pearson’s correlation using each participant’s mean response speed because this does not take trial or item variability into account (Chen et al., 2021; Rouder & Haaf, 2019). Averaging over trials assumes that all trials and items have the same effect, and we know this is not the case (Alario et al., 2004; Baayen & Milin, 2010). This procedure can therefore underestimate reliability (Chen et al., 2021; Haines et al., 2020; Rouder & Haaf, 2019). We chose to use the Bayesian framework rather than the frequentist framework because Bayesian analyses are better suited to estimating the precision of an effect. We can also obtain correlations of random effects in a frequentist hierarchical model; however, frequentist models only give us a point estimate of the correlation, whereas Bayesian models estimate a distribution of the correlation and a 95% credible interval. This allows us to better characterize the uncertainty of the estimate, which, as we explain in more detail in the next section, is crucial for making decisions about whether a measure is reliable enough for a given purpose. For example, a correlation of .7 would be indicative of good reliability by some standards; however, if the credible interval obtained for that estimate is wide (e.g., [.4,1]), that suggests that the true correlation could potentially be much lower and would no longer be considered to show good reliability.

We followed the procedure detailed in Chen et al. (2021) to fit the following models: To estimate split-half reliability, we fit a no-intercept model that predicts response times with a fixed effect of trial type (even or odd). Instead of estimating an intercept and slope for trial type, this model estimates intercepts for each level of trial type separately. The same structure was reflected in the by-participant random effects: we estimated by-participant intercepts for each level of trial type and random intercepts for item. This means that the correlation of the random by-participant adjustments indexes the correlation between the even and odd trials (i.e., split-half reliability). We repeated this process for the second session in a second model. To estimate test-retest reliability, we fit a third model that is identical to the one described here, except the fixed effect was session (first or second).

We used the following regularizing priors to constrain the model estimates so that extreme values will be unlikely (Schad, Betancourt, & Vasishth, 2021). For the intercepts, we assumed a normal distribution with a mean of 6.75 and a standard deviation of 1.5 on the log scale, which corresponds to a mean of 854 on the millisecond scale. One standard deviation below the mean would be 191 ms (exp(6.75)/exp(1.5)), and one standard deviation above the mean would be 3828 ms (exp(6.75)*exp(1.5)). For the residual error and the by-subject standard deviation, we assumed a truncated normal distribution with a mean of 0 and standard deviation of 1, and for the correlation between random effects, we used the LKJ prior with parameter *η* = 2. Below we report means and 95% credible intervals of the posterior distribution of the correlation between the by-participant random effects.

These analyses will serve as a conceptual replication of previous work that has shown that picture naming speed is reliable within an experimental session (e.g., Shao et al., 2012 and our pilot data mentioned above), and we expect to replicate this finding. Calculating split-half reliability will help validate the current stimulus set and procedure in order to calculate test-retest reliability. Split-half reliability can additionally be useful in interpreting test-retest reliability because estimates of split-half reliability serve as upper limits to the estimates we can expect to see for test-retest reliability.

As discussed in the introduction, there is no standard threshold that is used to determine whether a measure is reliable or not (e.g., Parsons et al., 2019), likely because what is considered “reliable enough” will depend on the purpose of the measure. It is of theoretical interest to know how reliable word production measures are over time, so to assess this reliability (both for split-half and test-retest reliability), we used a graded approach to interpreting correlation coefficients. The ranges of correlation coefficients and typical interpretations (Hedge et al., 2018; Landis & Koch, 1977) can be found in Table 2. To determine whether our estimates fall within or above the pre-defined ranges in Table 2, we used the region of practical equivalence (ROPE) procedure, as explained in Kruschke (2018). The ROPE procedure is a decision-making procedure, in which the researcher defines a range of values (the ROPE) that are “practically equivalent” to a value, such as zero (e.g., in a null-hypothesis significance test). The mean of the posterior distribution and 95% credible interval are computed, and if the credible interval falls completely within the ROPE, we accept that the data are “practically equivalent” to the target value (or range of values); if the credible interval falls completely outside the ROPE, we reject it. For the present purposes, we have defined a ROPE for each of the ranges in Table 2. If the credible interval falls completely within a certain ROPE, we will accept that range of values and interpretation; however, if it spans more than one ROPE, we will only accept that the measure has at least the reliability of the lowest ROPE that the credible interval spans. For example, if our credible interval falls completely within the ROPE for “excellent” reliability, we will accept that the reliability of the measure is excellent. If, however, the posterior mean is .82 but the credible interval is [.75,.89], we would only consider the measure to have “good” reliability because the credible interval overlaps with that ROPE.

**Table 2:** Ranges of correlation coefficients and their typical interpretations for reliability (e.g., Hedge et al., 2018; Landis & Koch, 1977).


CORRELATION COEFFICIENT	INTERPRETATION

.81–1	Excellent

.61–.8	Good

.41–.6	Moderate

<.4	Poor


We acknowledge that these are arbitrary ranges; however, they can be useful in deciding whether a measure is reliable enough for various purposes. In any case, we encourage readers to examine the posterior distribution means and credible intervals and decide for themselves whether these measures are sufficiently reliable for their purposes.

### Results

In the first session, the mean accuracy for all participants was 0.95, *SD* = 0.04. In Session 2, it was 0.96, *SD* = 0.03. The high accuracy rates for both sessions suggest that participants were indeed doing the task in good faith (Fairs & Strijkers, 2021).

**Figure 2:**
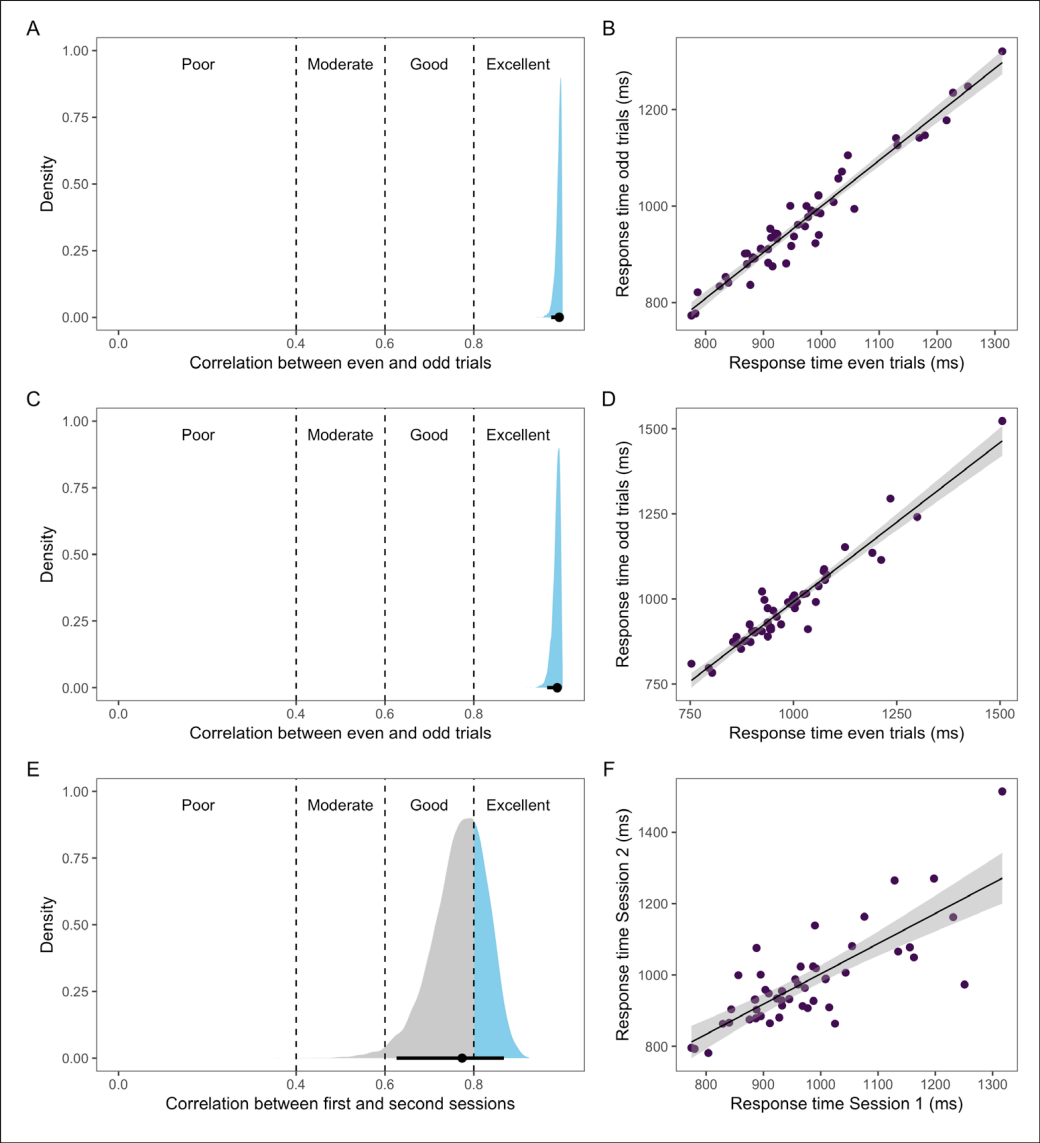
Posterior distribution with 95% credible interval (black line) (left side) and scatterplots with raw response time means (right side) for split-half reliability in Session 1 **(A-B)**, split-half reliability in Session 2 **(C-D)**, and test-retest reliability **(E-F)**.

The split-half reliability (i.e., correlation of response times in even and odd trials) in Session 1 was *ρ* = 0.99 [0.98, 1], and we replicate this high correlation in Session 2, *ρ* = 0.99 [0.97, 1] (see Figure 2). The test-retest reliability for Experiment 1 (i.e., the correlation of response times between Sessions 1 and 2) was *ρ* = 0.77 [0.64, 0.88] (see Figure 2).

### Discussion

In Experiment 1, our goal was to replicate findings previously reported in the literature on split-half reliability of picture naming speed, as well as to extend these findings and report test-retest reliability for these measures. Our results first suggest that the reliability of participants’ picture naming speed within a session (split-half reliability) is excellent: the correlation between the even and odd trials was almost perfect, and this high correlation was replicated in the second session. This finding is in line with at least one previous study conducted in the lab (Fuhrmeister et al., 2022) and with a recent study from our lab using on-line data (Bürki & Vasishth, in prep.). Good within-session reliability is necessary but not sufficient to claim that naming latencies reflect an intrinsic property or capacity of participants. It could be that this reliability reflects their motivational state or fatigue, or the pace they settle on for a given session.

The test-retest reliability in Experiment 1 provides information on how consistent participants’ picture naming speed was in the same task but at different points in time. Test-retest reliability was not quite as high as split-half reliability, but the correlation and credible interval still fell within the “good” range in our pre-defined ranges. This means that participants are fairly consistent in their picture naming speed even when tested again up to two weeks later, at least when performing the same task. The task was identical between the two sessions and participants had plenty of time (a total of three seconds) to name the pictures on each trial. As a result, test-retest reliability does not tell us whether some participants were slower because they were incapable of naming pictures faster, or whether they were slower because they chose a strategy (perhaps to prioritize accuracy) that led them to name the pictures slower. We address these possibilities further in Experiment 2.

## Experiment 2

In Experiment 2, we ask whether inter-individual differences in naming speed are consistent across different versions of a naming task, and we examine the possibility that participants are engaging in strategies to determine their speed of picture naming. To this end, participants completed a picture naming task under different conditions. We manipulated task condition by prompting participants to respond under time pressure in one condition (a speeded condition), and in a non-speeded condition, participants had the same amount of time to respond as in Experiment 1.

Split-half reliability in the non-speeded condition will serve as a replication of Experiment 1, and split-half reliability in the speeded condition can inform the interpretation of the correlation between task conditions. For instance, if this correlation is low or lower than the correlation between sessions in Experiment 1, split-half reliability estimates of *each* condition can suggest whether the correlation *between* conditions is low due to measurement error (i.e., low split-half reliability in one or both task conditions) or because participants are not consistent across different task conditions. The correlation of participant speed *between* task conditions can shed light on whether picture naming speed may be an intrinsic property or ability of participants or whether it reflects participants’ use of timing strategies in a given experimental context. For example, if we see a strong positive correlation between conditions, the strategy explanation would be less plausible because participants would still be ranked similarly by speed even when they do not have enough time to choose their pace.

One obvious strategy that participants could engage in is a speed-accuracy trade-off. To assess this specific possibility, we additionally correlated participants’ error rates with speed in each task condition separately to assess (in either condition) whether participants who respond faster are sacrificing accuracy to accomplish this.

### Methods

#### Participants

Sixty participants were recruited from Prolific (to obtain usable data from 50) with the same exclusionary and replacement criteria as in Experiment 1. Participants who participated in Experiment 1 were excluded from participating in Experiment 2. Participants’ data were excluded from the analyses if they did not return for the second session (*n* = 4), did not get at least 60% accuracy on the task (*n* = 2), or the data was not saved due to a technical error (*n* = 4).

#### Stimuli

The same stimuli from Experiment 1 were used for Experiment 2.

#### Procedure

**Figure 3:**
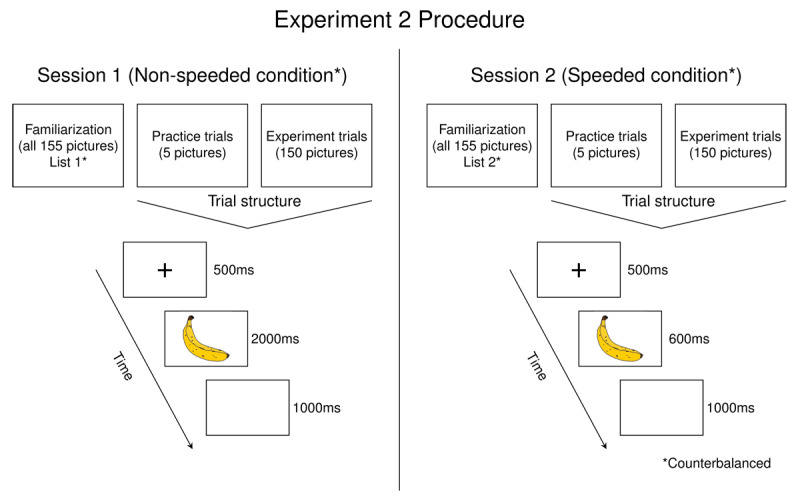
Illustration of procedure for Experiment 2. Participants completed the depicted experiment at two different sessions, each with a different stimulus list and condition (speeded or non-speeded). The order of the stimulus list presentation and the session at which participants receive the speeded or non-speeded condition was counterbalanced.

The experiment was conducted in two separate sessions that took place between 7 and 14 days apart (see Figure 3). Participants completed a simple picture naming task in both sessions. They completed the picture naming task under two different conditions: speeded and non-speeded. Pilot data from our lab from a similar task suggested that the order of the speeded conditions within a session influences naming speed, in that participants who had the speeded block first were also faster to respond in subsequent blocks even when it was not necessary to. Therefore, participants received only one condition (speeded or non-speeded) in a session. The order of presentation of the conditions and the list of stimuli that participants name in a session/condition were counterbalanced.

##### Picture naming task

The non-speeded condition was identical to the task described in Experiment 1 with the exact same trial structure. In the speeded condition, participants completed a speeded deadline task (e.g., Damian & Dumay, 2007; Gerhand & Barry, 1999; Kello et al., 2000). This task was similar to the non-speeded task, but the duration of the picture presentation was shortened: Each trial began with a fixation cross that appeared in the center of the screen for 500 ms, followed by a picture which appeared for 600 ms. The picture then disappeared and participants saw a blank screen for 1000 ms. Participants were asked to respond before the picture disappeared. As in Experiment 1 and the non-speeded condition, participants first completed a familiarization phase, in which they saw all the pictures they would name for that session presented with their corresponding name. They then completed five practice trials to practice the process, and they named all 150 pictures presented in random order. Vocal responses were recorded from the picture onset until the end of the trial.

#### Planned analyses

##### Response time analyses

The data were preprocessed as described in Experiment 1. Incorrect responses were excluded for disfluencies (non-speeded condition: *n* = 69, 0.92% of all trials; speeded condition: *n* = 136, 1.81% of all trials), no response (non-speeded condition: *n* = 58, 0.77% of all trials; speeded condition: *n* = 253, 3.37% of all trials), and wrong words (non-speeded condition: *n* = 199, 2.65% of all trials; speeded condition: *n* = 290, 3.87% of all trials), and response speed was calculated as the time from the stimulus onset to the vocal onset. No outlier trials were removed. For computing split-half reliability and the correlation between the two task conditions, we used the same procedures described above in Experiment 1: The correlation between by-participant random effects was computed in a Bayesian hierarchical model. We used the same priors as in Experiment 1.

These results will first provide a replication of the split-half reliability of Experiment 1, and they will additionally tell us whether participants’ word production speed is reliable within a session when participants are under time pressure. The strength of the correlation between the two versions of the task will inform us on the degree to which participants are consistent in their speed across speed conditions.

##### Speed-accuracy tradeoff

The correlation between participants’ speed on each version of the task alone will not be sufficient to tell us whether participants are or are not engaging in strategies or picking a certain speed to name the pictures. To this end, we correlated participants’ speed and accuracy in each version of the task (i.e., computed one correlation for the speeded version and one correlation for the non-speeded version).

For an estimate of participant speed to enter into these correlations, we extracted by-participant intercepts from the model that estimated the correlation between the two sessions (i.e., each participant had two intercepts). For an estimate of participants’ accuracy, we fit a Bayesian hierarchical model with a binomial link that predicts accuracy (0 or 1) and extracted the by-participant random intercepts. Like the response time model, this model included a fixed effect for task condition (speeded or non-speeded) but estimated separate intercepts for the two task conditions. We again used regularizing priors. For the intercepts, we assumed a normal prior with mean 0 and standard deviation of 1.5 (on the log odds scale). For the by-subject standard deviation, we assumed a truncated normal distribution with mean 0 and standard deviation of 1, and for the correlation between random effects, we used the LKJ prior with parameter *η* = 2.

Correlations between accuracy and speed for each version of the task were computed using the BayesFactor package (Morey & Rouder, 2018). For the prior distribution of the correlation, *ρ*, we used regularizing priors with a shifted and scaled beta (3,3) distribution (to center the distribution around zero instead of .5, Ly, Verhagen, & Wagenmakers, 2016). This distribution gives more weight to values around zero and downweights extreme values (i.e., –1 or 1). We report the mean of the posterior distribution of *ρ* and 95% credible intervals.

### Results

Participants also named the pictures with high accuracy in this experiment (non-speeded condition: *M* = 0.96, *SD* = 0.06; speeded condition: *M* = 0.91, *SD* = 0.06). Even though participants’ accuracy in the speeded condition was unsurprisingly slightly lower, these accuracy rates suggest participants were doing the task in good faith in this experiment as well.

**Figure 4:**
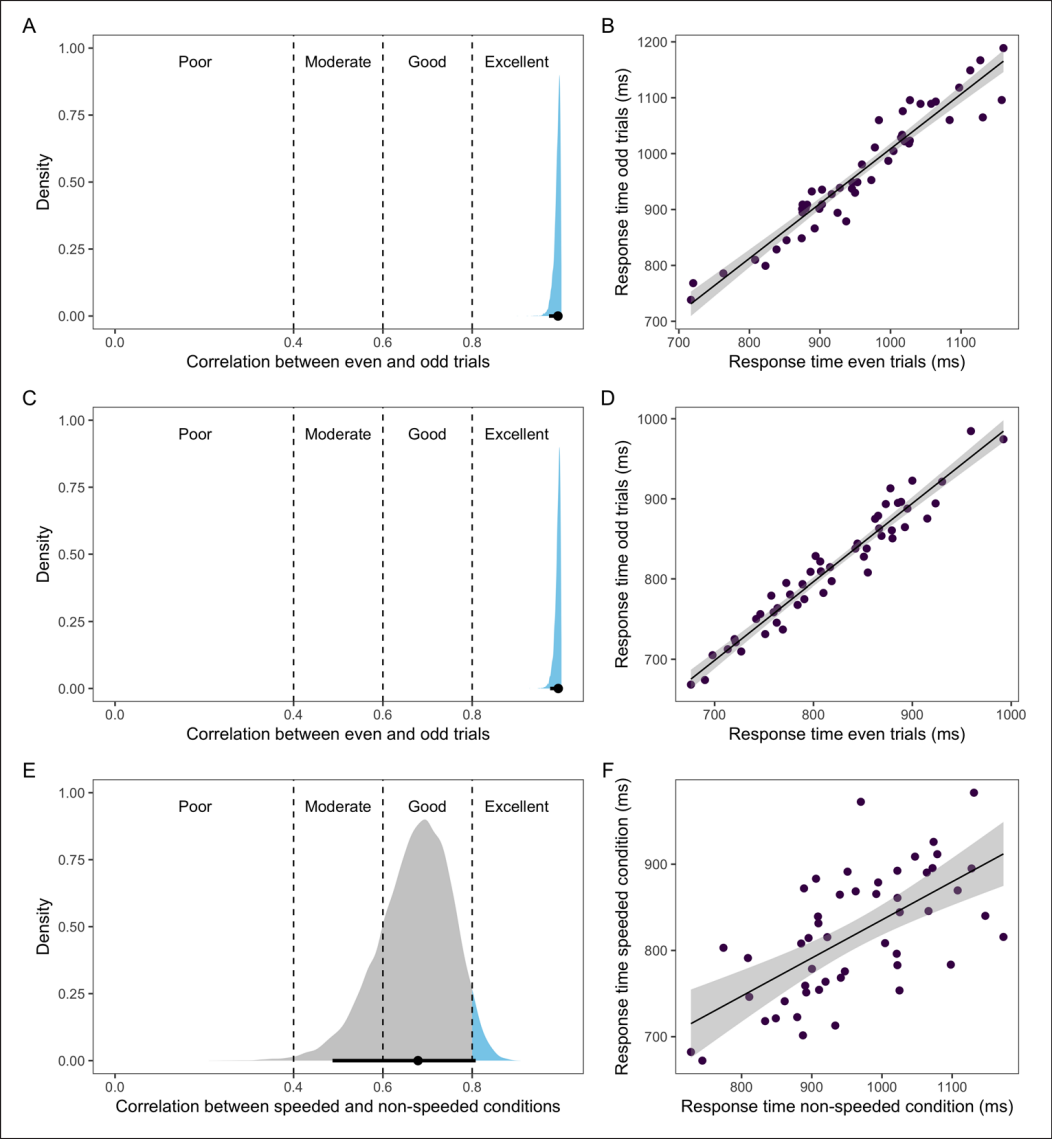
Posterior distribution with 95% credible interval (black line) (left side) and scatterplots with raw response time means (right side) for split-half reliability in the non-speeded condition **(A-B)**, split-half reliability in the speeded condition **(C-D)**, and the correlation between the conditions **(E-F)**.

The split-half reliability (correlation between even and odd trials) in the non-speeded condition was *ρ* = 0.99 [0.97, 1] and *ρ* = 0.99 [0.98, 1] in the speeded condition (see Figure 4). The reliability across conditions was *ρ* = 0.67 [0.51, 0.82] (see Figure 4).

**Figure 5:**
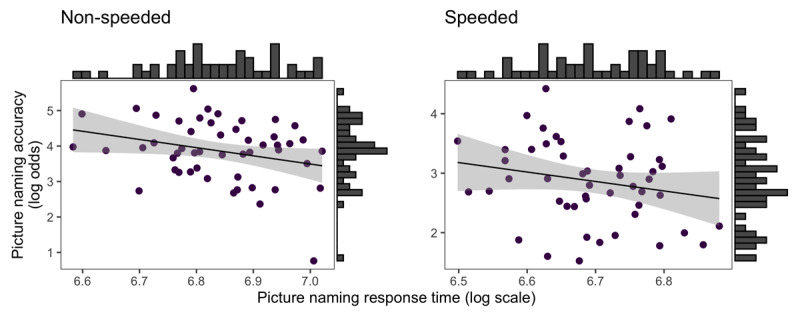
Scatterplots showing the relationship between participants’ picture naming speed in log units and accuracy in log odds in the non-speeded condition (left side) and speeded condition (right side).

To test whether participants were engaging in a speed-accuracy trade-off, we computed the correlation between participants’ accuracy and response speed in each condition separately. In the non-speeded condition, the correlation between accuracy and response time was *ρ* = –0.25 [–0.48, 0], and in the speeded condition, it was *ρ* = –0.17 [–0.41, 0.09] (see Figure 5).

### Discussion

We again replicated the excellent within-session reliability of picture naming speed in the non-speeded condition. The task participants performed in this condition was identical to the task from Experiment 1. We additionally found excellent within-session reliability in the speeded task, which suggests that participants are also very consistent in their naming speed within an experimental session when they are under time pressure.

The correlation between sessions in Experiment 2 was slightly lower than in Experiment 1 where the task was identical across sessions, and the lower bound of the credible interval only fell within the moderate range of our predetermined reliability ranges. However, a correlation of 0.67 is still fairly high. Participants who were faster in the non-speeded condition still tended to be faster even when under time pressure. This suggests that participants’ picture naming speed is at least somewhat consistent with slight task variations.

We additionally tested whether participants were engaging in a speed accuracy trade-off strategy. If this were the case, we would have expected a positive correlation between accuracy and speed (at least in the non-speeded condition) with longer response times associated with higher accuracy. Instead, we found negative correlations in both conditions, which is not indicative of a speed accuracy trade-off. In both conditions, faster participants tended to be more accurate at the task, suggesting that some participants are better at the task overall, both in terms of speed and accuracy. Note, however, that these correlations may not be very meaningful, especially considering that accuracy was almost at ceiling in both task versions.

## General discussion

Participants in word production experiments vary in their response speed, and it has been assumed that this variability can inform us on participants’ intrinsic capacities or on the properties of their language production system. For instance, several studies have tried to explain variability in picture naming speed with the participants’ performance on non-linguistic tasks, such as working memory or attention. Such an endeavor, in turn, assumes that picture naming latencies are reliable, i.e., consistent over trials, time, and (possibly) different naming tasks. The aim of the present study was specifically to assess this reliability.

Taken together, the results of Experiments 1 and 2 reveal that picture naming latencies are consistent over trials, time, and tasks. In other words, there are fast and slow participants, and fast participants tend to remain fast while slow participants tend to remain slow. At the theoretical level this means that naming speed could indeed be indicative of a property or capacity that is inherent to the individual. However, an important question for future studies will be to determine whether inter-individual variability in naming speed can be linked specifically to the language production system or whether it reflects a participant’s speed of initiating *any* response to a stimulus. In an experiment comparing younger and older participants, Boudiaf et al. (2018) reported an increase in response times in picture naming tasks as well as in several non-linguistic tasks with increasing age. Moreover, the increase in naming speed was no longer significant once the model accounted for participants’ speed on a numerical judgment task. These findings suggest that variability in naming speed (here between the two groups) is not specific to the language production system. Another recent study by Hintz et al. (2020) had young adults perform a picture naming and a lexical decision task, as well as a battery of tasks measuring general processing speed. They found a relationship between general processing speed and response times on both the picture naming and lexical decision tasks, suggesting that at least some of the variance in response speed on these tasks can be explained by domain-general processing speed. Of course, the question remains of whether this variability reflects a general processing speed of any response or the reliance of each of these tasks on the same available cognitive resources. In sum, the demonstration that a reasonable amount of between participant variability is reliable shows that the study of this variability can be informative. Ultimately, additional work will be necessary to determine the extent to which this variability can provide information on the language production system per se.

Despite the relatively high reliability we found in both experiments, we note that there is still a substantial amount of unexplained variance. The estimate of test-retest reliability in the first experiment was 0.77, which means that 41% of the variance was unexplained. The amount of unexplained variance in Experiment 2 was 55%. The question may arise of whether a portion of the unexplained variability in our study comes from the fact that we tested participants online rather than in the lab. Because of the online setting, we had less control over certain factors such as the hardware that participants used to perform the experiment or whether the participants were distracted by external stimuli. We do not necessarily think that the data would have been more reliable if we had collected them in the lab for two reasons. First, the accuracy rates were very high: the majority of participants scored over 90% accuracy in each session of each experiment, and this is similar to accuracy rates observed in lab settings. Moreover, split-half reliability for each session and task was almost perfect and similar to what we observe in lab data (e.g., Fuhrmeister et al., 2022). We therefore expect the amount of unexplained variability to be similar in a lab setting.

We originally chose to correlate even and odd trials within an experimental session to estimate split-half reliability; however, there are other ways to compute this, such as correlating trials from the first and second halves of the experiment. Given that trial order has been found to impact response speed in picture naming tasks (e.g., Gordon & Cheimariou, 2013), we may find that split-half reliability is lower when computed with the two halves of the data rather than with even vs. odd trials. Following a reviewer’s comment, we performed exploratory analyses (i.e., these analyses were not pre-registered) to estimate the split-half reliability for each session/condition in each experiment using the trials from the first and second halves of the session rather than even and odd trials. The results for these analyses can be found in Table 3. We did find slightly lower split-half reliability when computing the estimates with the first and second halves of the data; however, all estimates and lower bounds of the credible intervals except for one (non-speeded condition of Experiment 2) still fell within the excellent range.

**Table 3:** Posterior distribution means and credible intervals for split-half reliability calculated with first and second halves of the data.


MODEL	ESTIMATE	CI_LOWER	CI_UPPER

Experiment 1 Session 1	0.93	0.88	0.97

Experiment 1 Session 2	0.95	0.91	0.98

Experiment 2 Non-speeded	0.87	0.77	0.94

Experiment 2 Speeded	0.94	0.89	0.98


On a methodological note, we acknowledge that the estimates reported here would likely have been more precise (i.e., had a narrower credible interval) if we had collected data from a larger sample size. This was unfortunately not possible due to the amount of manual labor involved in pre-processing the data. The Bayesian credible intervals reported here inform us on the most likely values of the correlations and the uncertainty of these estimates. In our interpretations, we were careful to consider the entire range of the credible interval to ensure our interpretations were not too optimistic. For example, the mean of the posterior distribution for the correlation between speed conditions in Experiment 2 fell within the pre-defined range for “good” reliability; however, the lower bound of the credible interval fell within the “moderate” range. We therefore only considered the measures in Experiment 2 to have moderate reliability because the values close to the lower bound of the credible interval are also likely values.

Nonetheless, the findings of the present study have important methodological implications. As explained in the introduction, the sample size required to detect a correlation between two measures partly depends on the reliability of each of these measures. The reliability estimates reported here can be used to calculate sample sizes that we would need to detect correlations of various magnitudes between picture naming speed and other measures, given that we know the reliability of those measures as well. As an example, we will calculate the sample size needed to detect correlations between a simple picture naming task and the operation span task, which is a common task used to measure working memory (e.g., Conway et al., 2005). Klein and Fiss (1999) tested participants on this task several times and report test-retest reliability of *r* = .73 between the first two times the task was administered with three weeks between the tests. For picture naming, we will use the estimate of test-retest reliability from Experiment 1 (0.77). If the true correlation between a picture naming measure and the operation span task is .4, we would need 84 participants to detect this correlation with 80% power at an alpha level of 0.05. For a true correlation of .3, we would need 152 participants, and for a true correlation of .2, we would need 346 participants.^4^ This suggests that we may need much larger sample sizes than what is typical in this literature if we want to detect correlations between measures of picture naming speed and other cognitive measures.

What do the current findings mean for the existing literature that has tried to explain individual variability in picture naming speed by correlating naming latencies with various measures of cognitive abilities? As we saw above, unless the correlations we can expect to see between these variables are large (which is unlikely), many existing studies (including our own) may have had sample sizes that were too small to detect these correlations. Especially in studies of language production, small samples may be unavoidable because of the amount of manual labor involved in setting the onset of the vocal response on each trial. Unfortunately, however, this means that studies relying on such correlations may be especially susceptible to Type II errors. If these types of studies are underpowered, there is also the possibility that significant correlations that are reported are actually overestimates of the true effect (i.e., Type M errors, see Gelman & Carlin, 2014).

To conclude, we report test-retest reliability estimates of picture naming speed for the first time. Our findings show that inter-individual variability is reliable and can therefore be informative of the language production system or non-linguistic abilities. We hope that the reliability estimates reported here will be useful for other researchers carrying out correlational studies on inter-individual differences in language production (e.g., to calculate sample sizes for such designs).

## Footnotes

Imageability is also a robust predictor of naming latencies (Alario et al., 2004); however, we did not have imageability estimates for the pictures used for the present study.

As described in the next section, we computed the correlation between the two halves of the data or sessions in a hierarchical model. Due to shrinkage from the model, a few outliers should not influence the correlation very much.

Our ethics board does not allow us to make raw audio recordings of participants publicly available. However, the preprocessed data set includes the raw output from the experimental software (participant number, trial number, item), as well as the response time and accuracy for each trial.

Note that these sample sizes may be optimistic: If we computed them using the slightly lower estimate from Experiment 2 (*r* = .67), we would need 97 participants to detect a true correlation of .4, 175 for a correlation of .3, and 398 for a correlation of .2.

## Appendix A

**Table:**

LIST 1	LIST 2

anchor	airport

apple	ambulance

arm	ankle

arrow	apron

avocado	asparagus

basket	baby

battery	back

beak	balloon

bear	banana

bell	bat

bench	beard

bib	bed

bike	bedroom

bone	belt

book	bible

bottle	bin

boxer	bomb

bracelet	boomerang

bull	box

bullet	bra

bus	brain

butter	broccoli

butterfly	broom

candle	burger

carrot	butcher

cherry	button

clown	cactus

coat	cage

comb	calculator

compass	calendar

computer	camera

cork	cannon

crab	car

dice	caravan

doctor	chain

donkey	chair

doughnut	chocolate

drawer	choir

dress	cigarette

drum	cloud

ear	coconut

elbow	coffee

fairy	coffin

fireplace	condom

flag	cone

flower	cowboy

fox	crown

fridge	dentist

ghost	desert

glove	devil

heel	diamond

helmet	dinosaur

island	dog

jellyfish	dolphin

kangaroo	dominoes

kite	door

kiwi	dragon

lawnmower	duck

lighter	earring

limo	egg

magnet	elephant

map	eye

medal	face

mermaid	fan

net	farm

peg	feather

plumber	finger

pocket	fire

rabbit	fist

rhino	foot

rocket	football

shield	frog

shower	fruit

tomato	giraffe

tray	girl

lung	glass

castle	glasses

hippo	greenhouse

tractor	guitar

circle	gun

skirt	hair

mouth	hand

dummy	handle

camel	harp

olive	heart

robot	hedgehog

tie	honey

knife	house

snake	iron

onion	jar

thumb	keyboard

cow	king

squirrel	knee

piano	lab

sun	leaf

suit	leg

goat	lemon

pipe	lion

lamp	lizard

caterpillar	man

key	mask

suitcase	maze

owl	microphone

curtain	mirror

saw	moustache

kitchen	needle

flipper	nose

rope	nun

koala	nurse

lighthouse	parachute

wall	parrot

fence	penguin

shoe	pilot

potato	pirate

teacher	pizza

fork	pool

gym	printer

helicopter	pumpkin

hat	ring

wing	road

whale	roof

pear	scarecrow

soap	scarf

mountain	scorpion

zebra	screwdriver

sweet	shadow

sausage	shark

wave	sheep

tattoo	shirt

submarine	sink

triangle	skateboard

tunnel	skeleton

star	skull

tongue	snail

sword	sock

judge	spider

pencil	spoon

orange	stapler

rose	strawberry

nest	swimming

puppet	tank

train	telephone

queen	television

pepper	thermometer

pineapple	tooth

tiger	torch

scissors	trumpet

ruler	umbrella

windmill	vein

wheelbarrow	volcano

tap	waiter

swan	wallet

tambourine	watch

zip	well

tree	witch
